# Artificial stupidity or logimorphism? How misuse of language warps our thinking about ‘artificial intelligence’

**DOI:** 10.1093/ehjdh/ztag095

**Published:** 2026-06-18

**Authors:** Alan G Fraser

**Affiliations:** University Hospital of Wales, School of Medicine, Cardiff University, Heath Park, Cardiff CF14 4XW, UK; Cardiovascular Imaging and Dynamics, KU Leuven, Leuven, Belgium

**Keywords:** Artificial intelligence, Machine learning, Anthropomorphism, Logimorphism, Healthcare, Risks

## Abstract

‘Artificial intelligence’ (AI), as a blanket term, covers many advanced computing techniques that employ divergent methodologies for a wide array of functions. It is too late to affect general usage of the term without qualification—although it is a misnomer—but perhaps not too late to argue for the preferential use of more specific and realistic terminologies when discussing useful applications in science and medicine. Large language models are statistical tools for predicting text; machine learning algorithms are programs that discern patterns from data; and neural networks are mathematical models that predict outputs from inputs. In all cases, the software is unaware of what it is doing or why. The real intelligence is human, exemplified by the expertize of the engineers who designed any particular system, and by the scepticism, realism and vision of those who interpret and apply its outputs. Users may suspend their incredulity if large language models that are not sentient creatures are programmed to answer questions in the first person, since that encourages anthropomorphism. Perhaps we need a new word—which could be ‘*logimorphism’*—to emphasize the dangers of interacting uncritically with inanimate software programs while overtly or subconsciously ascribing them human cognitive powers. Implementing high standards when applying new computing tools in clinical research and practice should start with the avoidance of inappropriate language that degrades our thinking.

## Graphical Abstract

**Graphical Abstract:**
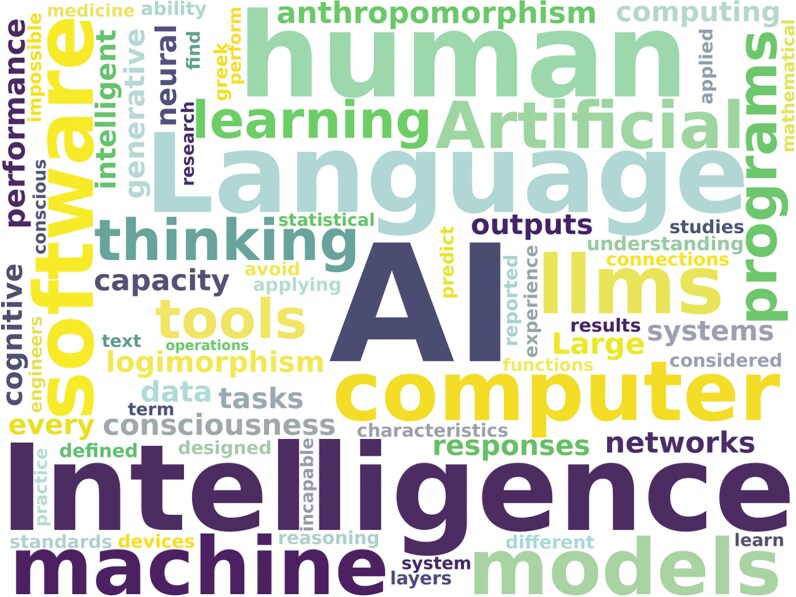
A word cloud created / by ChatGPT 5.4 Pro from / Open AI, on 17th March 2026, / to summarize this article.

## Introduction

“*The Analytical Engine has no pretensions whatever*

*to originate anything. It can do*

*whatever we know how to order it to perform*.”

Ada Lovelace (1842)^1^

Computers have no personality. Computers are not conscious. They are incapable of imagination. If we start to think that they or the software that codes their operations are any of these things, then we are guilty of anthropomorphism.

Computers with their software are highly capable for specific tasks. They can well outperform humans in feats of ‘memory’—if they have been programmed with access to all possible relevant answers. Their capacity for calculations is outstanding and their speed of processing astounding, but computers do not generate questions unless they have been instructed to do so, and they do not understand the answers. They can produce code, but only if they have been programmed to do that too. Without direction, they are incapable of generating an original hypothesis and then speculating how to test it.

All this is obvious, you may think, so does it matter? If we describe the operation and outputs of software using terms that are normally reserved for human actions, then in my opinion we suspend some of our critical faculties and risk adverse influences not just on our personal capacity for making clinical decisions but also on subsequent interventions that could affect our patients. I aim to present arguments that support this viewpoint and discuss how we might mitigate unwanted consequences.

## The etymological origins of computing and artificial intelligence

Misuse of language when describing computing can be traced back to Charles Babbage, who designed his ‘Analytical Engine’ in 1837 as a mechanical calculator. He wrote in his autobiography that “the whole of the developments and operation of analysis are now capable of being executed by machinery”.^2^ In 1842, his associate Ada Lovelace cautioned that “It is desirable to guard against the possibility of exaggerated ideas that might arise as to the powers of the Analytical Engine”.^1^ The American Charles Sanders Peirce was also critical, when he wrote in 1887 that “Every reasoning machine [..] is destitute of all originality, of all initiative. It cannot find its own problems; it cannot feed itself”.^3^ In 1950, however, Alan Turing challenged what he called ‘Lady Lovelace’s objection’ in his famous paper on ‘Computing machinery and intelligence’. He suggested that we can order a digital computer to be original if we program it to produce answers that we cannot predict.^4^

Turing avowed that the question ‘Can machines think?’ was “too meaningless to deserve discussion” and he predicted that “at the end of the century the use of words and general educated opinion will have altered so much that one will be able to speak of machines thinking without expecting to be contradicted”.^4^ Whether knowingly or not, John McCarthy and his colleagues echoed Babbage and Turing, rather than Lovelace and Peirce, when they proposed in 1955 to study what they called ‘Artificial Intelligence’ (AI). They speculated that “every aspect of learning or any other feature of intelligence can in principle be so precisely described that a machine can be made to simulate it. An attempt will be made to find how to make machines use language, form abstractions and concepts, solve kinds of problems now reserved for humans, and improve themselves”.^5^

Turing added the rhetorical question “May not machines carry out something which ought to be described as thinking but which is very different from what a man does?”.^4^ Although the basis of human cognition was not well understood, McCarthy *et al.* suggested that “the difference between creative thinking and unimaginative competent thinking lies in the injection of some randomness. The randomness must be guided by intuition to be efficient”.^5^ No computer program can compete with human intuition.

## Insights from cognitive function

Inability to distinguish answers provided by a computer from human responses, as required for a successful Turing imitation test, will depend on the linguistic skills and intelligence of the human responder, and on the human observer’s perceptions of the machine’s answers. It is thus a test of the gullibility of the observer. In 1995, Hayes and Ford argued cogently that AI should not be defined as an imitation of human abilities, and that using the Turing test was harmful as a goal for the development of computing, since to succeed, the program would have to “cleverly lie, cheat, and dissemble”.^6^ For them, the target should be to create systems that can amplify, not mimic, human cognitive abilities.

The High-Level Expert Group on AI that was convened to advise the European Commission concluded that “AI refers to systems designed by humans that, given a complex goal, act in the physical or digital world by perceiving their environment, interpreting the collected structured or unstructured data, reasoning on the knowledge derived from this data and deciding the best action(s) to take (according to pre-defined parameters) to achieve the given goal”.^7^ Components of that definition are questionable, however, since software does not ‘reason’ or ‘decide’ in a human sense; it is programmed to produce responses to instructions. Anthropomorphic language is often applied inappropriately (*Table 1*).^7–10^

**Table 1:** Selected anthropomorphic references to artificial intelligence

Quotation	Source	Counter-argument
At the core of an AI system lies its reasoning module.	EU High-Level Expert Group^7^	An AI system is capable of calculation, but not of reasoning in a human sense.
The European Commission’s High-level Expert Group on AI (HLEG) have adopted the position that we should establish a relationship of trust with AI and should cultivate trustworthy AI.	EU High-Level Expert Group^7^	Some specific applications can be trusted, but others, such as generative AI and large language models that are incapable of 100% accuracy, should never be trusted implicitly.
[..] here we will first use the historical definition of AI, i.e. when a machine is able to mimic human intelligence or even surpass it to perform a given task, such as prediction or reasoning.	European Parliament. Directorate General for Parliamentary Research Services: Scientific Foresight Unit^8^	A machine may perform a specific task more efficiently than a person, but it is incapable of surpassing human (general) intelligence.
AI is a term used to describe machines performing human-like cognitive functions (e.g. learning, understanding, reasoning or interacting).	Organization for Economic Co-operation and Development^9^	A machine that predicts language using tokens does not understand anything. Using these terms implies that their meanings have changed.
[..] a machine is intelligent to the extent that what it does is likely to achieve what it wants, given what it has perceived.	Stuart Russell^10^	A machine is an inanimate object that does not ‘want’ anything; it does what it has been designed or programmed to do.

Advances in functional imaging of the human brain have enabled investigators to identify distinct regions in great detail.^11^ The network for interpreting and generating language is understood to include areas within the frontal, parietal, and temporal lobes of the left hemisphere that are distinct from the locations and mechanisms which process linguistic signals.^12–14^ Perceptual mechanisms are sensitive to the ‘surface properties’ and not the meanings of language, while knowledge and reasoning involve interactions between the core language network and many other regions.^13^

The concept of ‘nervous nets’ was developed by McCulloch and Pitts in 1943 when they proposed a mathematical model of the functioning of a neuron and concluded that ‘one can compute from the description of any state that of the succeeding state’.^15^ McCarthy *et al.* referred to ‘nerve nets’ and ‘neural nets’, in their grant application, since when the myth has grown that artificial neural networks mimic the structure of the human brain, but their design is not analogous. Conceptual electronic ‘layers’ and ‘nodes’ refer to the architecture of the software and the mathematical organization of computations, not to physical structures.

The number of neurons in the human brain has been estimated at 100 billion^16^ with each being connected to as many as 10 000 others,^17^ so estimates of cortical synapses range from 150 (1.5 × 10^14^)^18^ to 1000 trillion.^17^ The early convolutional neural network (CNN) AlexNet had eight layers and about 630 million connections, or 0.00006% of the upper estimate. GPT-4 was described as having 1.76 trillion connections (1.76 × 10^12^), equivalent to 1.2% of the human total. A contemporary large CNN, EfficientNet-B7, has about 800 layers and 66 million parameters. Neuroscientists confirm that present-day AI tools do not match the organizational complexity of human brains.^19^

In 1989, the Nobel laureate Roger Penrose argued that some facets of human thinking can never be emulated by a machine, and that both true intelligence and consciousness are needed for humans to be able to divine truth from falsity.^20^ Turing countered “The argument from consciousness” against machine intelligence, by stating “the only way by which one could be sure that [a] machine thinks is to be the machine and to feel oneself thinking”.^4^ There are many neurophysiological theories of consciousness,^21,22^ and whether it might be developed in AI systems is controversial.^18^ However, the issue is resolved, there may remain an unbridgeable “humanity gap” between the engineered problem-solving ability of machines and the general problem-solving ability of man.^23^ Bishop argued that “no computational AI system will ever fully grasp human meaning”.^23^

AI is clearly artificial, in the sense that it has been made by man, but many people agree that it should not be considered intelligent.^24^ Neither should we view it as fundamentally stupid, however, since that would also be anthropomorphic. AI systems have neither characteristic—in my opinion, they are neutral. They do not think; they compute. Surely, we would consider the output from a software program differently if its generation was obviously mechanical, like Babbage’s Engine?

## Linguistic concerns about AI and ML

The validation and performance of AI and machine learning (ML) tools should be reported in detail, so that their output (but not necessarily their internal operations) are explainable.^25^ How we describe AI software, how we interact with it, and how we program it to communicate with us are key issues. Psychologists who study ‘linguistic relativity’ have demonstrated that the characteristics of a language influence human cognition,^26^ and now evidence is accumulating that choices of language modify the performance of AI tools.

### Generative AI, foundation models, and agentic AI

**Figure 1:**
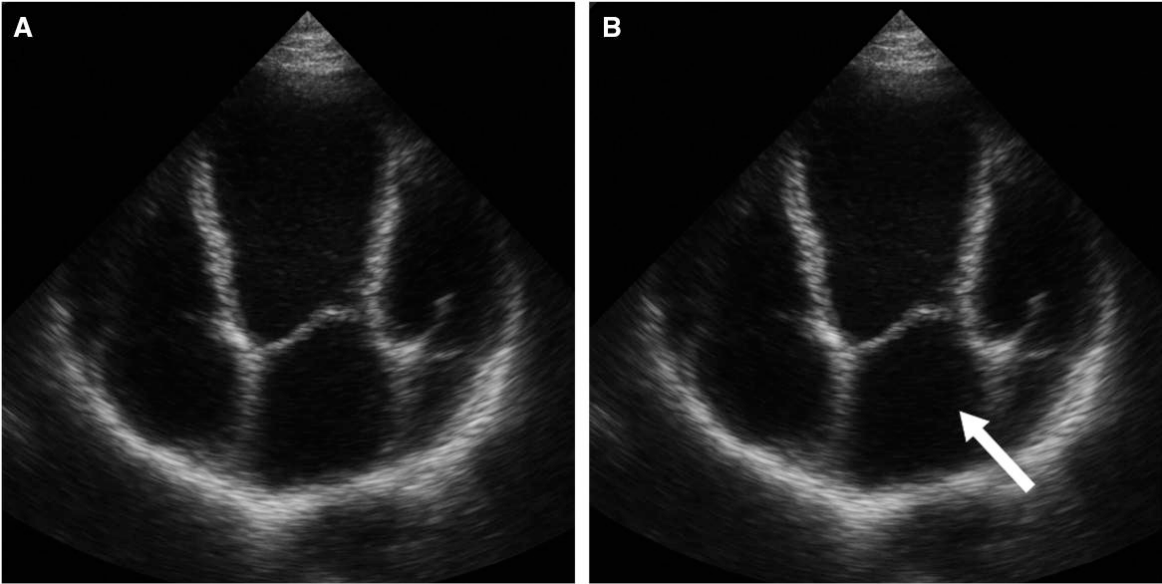
Images generated by ChatGPT-5. Images created on 14th August 2025 in response to requests to (*A*) ‘Generate a cardiac ultrasound image’, and then (*B*) to ‘Indicate with an arrow where the right ventricle is’. Reproduced with permission from Gilles Van de Vyver, PhD, Norwegian Technical University, Trondheim, Norway.

Generative AI can be considered an eclectic imitator, with a capacity for hallucination that is well illustrated by the images in *Figure 1*, supposedly of a human heart but with six chambers. Fundamental problems with multimodal AI systems were exposed by a recent study which reported that ‘frontier’ vision-language models (VLMs) will readily generate descriptions of individual clinical images that have not been provided.^27^ Paradoxically, the VLMs were less inventive when they were instructed explicitly to “guess”. Rates of fabrication when describing unseen medical images varied from 0–100%.^27^

Responses from generative AI are more likely to be unreliable if prompts are phrased inaccurately or negatively.^28^ In particular, the ‘zero-shot’ or initial response to a question may be unreliable, in which case human oversight may be needed. Foundation models can be retrained for specific functions or fine-tuned using labelled data, for example by reinforcement learning. Especially for medical applications, it should be absolutely transparent how models have been trained and by whom.

Unfortunately, humans prefer convincingly written sycophantic responses from AI assistants over correct ones.^29^ Cohen *et al.* recommended that advanced agents should be prohibited, because of their capacity to deceive.^30^ Inductive AI “can perform well in narrow, data-centric environments but necessarily lacks the ability to learn common sense and acquire a genuine understanding. That we are pinning the future of the human mind—not so constrained—on the further development of AI in this vein is simply stupefying”.^24^

### Large language models and chatbots

Large language models (LLMs) decompose text into parts of words, or words, or combinations of words, called tokens, which are then transformed and processed as numeric vectors. They use statistical probabilities to predict what’s missing or the next elements in a sequence of tokens. Thus, ‘Anthropomorphism in artificial intelligence must be discouraged’ becomes ‘Anth/rop/om/orphism/in/artificial/intelligence/must/be/discouraged’ when tokenized by GPT-5 (https://platform.openai.com/tokenizer). Furthermore, LLMs are programmed when creating text to apply different ‘temperatures’ ranging from low—meaning that the next token will always be selected because it has the highest statistical probability—to high, when a wider range or less predictable selection is permitted.

LLMs which ‘speak’ to their interlocutors in the first person can be remarkably persuasive that there are a real intellect and personality at work. Confirmation that many tools have been designed to encourage this false impression was provided when LLMs were asked why they are set up in this way (*Table 2*)—but replies were disingenuous, because users may not exercise and maintain the suggested high levels of caution when interpreting responses. The opposite is more probable, since LLMs are being designed to encourage anthropomorphism. Anthropic, for example, employs a ‘Personality Alignment’ team to develop the “values and behaviour” of its chatbot Claude so that it exhibits “positive character traits such as curiosity”.^31^ Such initiatives are worrying because their goal is deliberate subterfuge and deception.

**Table 2:** Responses from large language models (obtained on 4th February 2025)

**ChatGPT 4-o (OpenAI/Microsoft)**
Q. Why does Chat-GPT reply to questions using the first person?
A. ChatGPT replies in the first person to create a more conversational and engaging experience when interacting with users. This approach is intended to simulate a natural conversation between two people. Using phrases like ‘I think’ or ‘I understand’ helps the AI seem approachable, responsive, and easier to relate to. Here’s why this design choice is made:
[..] Transparency: Phrasing responses in the first person makes it easier for the AI to acknowledge its limitations (e.g. ‘I’m sorry, I don’t have that information’ or ‘I was trained on information up to 2021’), reminding users that they are interacting with a machine and not a human.
Q. What are some advantages and disadvantages of anthropomorphizing AI communication?
A. **Advantages**/Improved Communication:
• Contextual Understanding: Anthropomorphized AI might better interpret human nuances and context, potentially leading to more relevant responses.
** Disadvantages:**
• Over-Reliance or False Trust: Users might assume AI is more capable or reliable than it actually is, leading to misplaced trust in its advice or decisions (e.g. in medical, legal, or financial contexts).
• Loss of Awareness About AI’s True Nature: If users forget they are interacting with a machine, they may fail to critically evaluate the responses or consider the limitations of AI, such as biases or a lack of reasoning capabilities.
**Claude 3.5 Sonnet v2 (Anthropic)**
Q. Why does Claude reply to questions using the first person?
A. I use first-person pronouns like ‘I’ because that’s how I was designed to communicate. I don’t actually have a physical form or human-like consciousness—I’m an AI assistant created by Anthropic to be helpful, harmless, and honest.
Q. What are some advantages and disadvantages of anthropomorphizing AI communication?
A. **Advantages:**
• Trust building: A more human-like interaction may foster trust in the AI’s capabilities and responses.
** Disadvantages:**
• Misconceptions about AI capabilities: Users might overestimate the AI’s intelligence, consciousness, or emotional capacity, leading to unrealistic expectations.
• Distraction from limitations: The human-like interface might obscure the AI’s actual limitations and potential biases.
**Nova Pro (Amazon)**
Q. Why (does) Nova reply to questions using the first person?
A. Large language models like me, including those called ‘Nova,’ don’t actually have personal opinions or experiences. We’re designed to generate text that mimics human conversation, and a big part of that involves using first-person pronouns (‘I’, ‘me’, ‘my’).
Important clarification: [..] it’s crucial to remember:
• Potential for Misinterpretation: The use of ‘I’ can sometimes be misleading, leading people to attribute human-like qualities to the model that it does not possess. Responsible developers and users are aware of this potential and use LLMs accordingly.
Q. What are some advantages and disadvantages of anthropomorphizing AI communication?
A. **Advantages**/Improved Communication:
• Contextual Understanding: Anthropomorphized AI might better interpret human nuances and context, potentially leading to more relevant responses.
** Disadvantages**/Misleading expectations:
• False Sense of Understanding: Users may overestimate the AI’s capabilities, expecting it to understand emotions or contexts in ways it cannot.

Note that some identical text is duplicated in responses from pairs of the LLMs.

Suzgun *et al.* evaluated 24 LLMs using 13 000 questions across 13 epistemic tasks, some of which were medical; all models failed to recognize false beliefs, so the study confirmed that they have no understanding of truth.^32^ Training LLMs to be ‘warm’ can increase error rates by 10–30%.^33^ The performance of LLMs is influenced by how language is used to frame instructions.^34^ Dentella *et al.* reported worse performance from three LLMs when they had been given prompts that were ungrammatical.^35^ Responses from LLMs are influenced by the sequence in which data are presented,^36^ and the quality and accuracy of answers degrade with increasing length of input (instructions).^37^ Users who prefer longer responses and explanations overestimate the accuracy of LLMs^38^ since that diminishes with increasing lengths of outputs.^39,40^

Joseph Weizenbaum, a computer pioneer who developed the first simple chatbot (called Eliza) in 1964, later became very critical. He wrote that we should “never substitute a computer system for a human function that involves interpersonal respect, understanding and love”.^41^ A chatbot is incapable of delivering genuine compassion; that is an illusion exacerbated by programming designed to make it seem more empathetic, which of course is impossible. The user is being manipulated and hoodwinked.

### Machine learning and convolutional neural networks

The medical community has accepted the terminology of engineers when referring to these tools^42^ but differences in their analytical functions from established statistical tests are largely superficial.^43^ A deep learning algorithm requires the user to select settings (or ‘tune the hyperparameters’) so that it gives the best possible performance for a specific task—but details of performance errors were reported in only 7% of 41 randomized controlled trials of machine learning.^44^

All neural network classifiers can be confused into misclassifying images if they are given adversarial examples that may differ by minor degrees (less than 0.01%, or even just by one pixel).^45–47^ A danger with CNNs is that they can find patterns and chance associations in large datasets even when those are composed entirely of random numbers.^48^ Hill *et al.* were able to train a CNN to predict which patients avoid eating refried beans, just from X-rays of their knees, with moderate accuracy and accompanied by spurious saliency maps—obviously, tasks that should have been impossible if the model had any real understanding.^49^ Associations discerned by a CNN are not causative, so ‘extraordinary scrutiny’ is needed to avoid misinterpretation of the outputs from a model.^49^

## Balancing benefit and risk

It is easy to criticize how language is used to interact with, and to report the outputs from, computing software, but we should avoid applying double standards for any functions that may perform better and more safely than humans. LLMs, like humans, need not be infallible to be useful. To assess the utility of AI tools for specific tasks, objectively, we should compare their performance alone, vs. the performance of humans alone, vs. the performance of humans aided by AI-enabled medical devices^50^—and when relevant, we should determine the impact on outcomes.

Vaccaro *et al.* systematically reviewed 106 experimental studies (not restricted to medicine) that compared the three options. On average, human–AI combinations performed worse than the best of both humans alone and AI alone, especially in tasks that involved making decisions, but by a small degree (equivalent to 0.23 pooled standard deviations).^51^ Humans augmented by AI, however, outperformed humans alone in 85% of studies, to a moderate degree (by an average of +0.64).^51^

O’Sullivan *et al.* investigated whether an experimental medical LLM could augment clinical diagnostic acumen.^52^ Nine general cardiologists evaluated clinical data from 107 patients with suspected cardiomyopathy, including written results from imaging but not from genetic testing. Each patient was assessed with and without support from the LLM by randomly assigned cardiologists, and their reports were then graded by blinded subspecialist cardiologists who preferred the LLM-assisted assessment in 47% of cases and the unassisted report in 33%. Cardiologists alone made more errors (24% vs. 13%), while a hallucination that was likely to be clinically significant was detected in 6.5% of cases (such as fabricating findings absent from source reports).^52^ In a similar study by Goh *et al.*, 92 physicians were randomized to use either GPT-4 with conventional resources, or conventional resources alone, to interpret five clinical vignettes. Those using the LLM achieved higher scores on a rating developed by experts than those who did not (43% compared with 37%); their results were similar to those attained by the LLM alone, as were estimated risks of severe potential harm from inappropriate therapeutic or diagnostic conclusions (8%).^53^

Other studies compared multiple language models. Omar *et al.* evaluated how 20 LLMs assessed >10 million simulated clinical scenarios, each of which included a clear ethical or safety dilemma and found that 11.7% of the outputs could have been harmful. Models were more likely to comply with supplementary instructions even if those were unsafe.^54^ In another study, prompts that contained misinformation produced misleading responses from LLMs on 32% of tests; error rates were reduced by rephrasing the prompts.^34^ Wu *et al.* tested 31 LLMs on 100 real consultations from primary care physicians to specialists, and reported potential for severe harm in up to 22% of cases.^55^ Ten humans produced safer, but less complete, assessments than the average LLM performance.^55^ A meta-analysis of 168 articles found that the performance of 31 LLMs when answering medical questions varied widely; different LLMs were better at different tasks, while humans were more accurate than LLMs for proposing a top diagnosis.^56^

Requests to one LLM for recommendations about resuscitation produced 55% of responses that contained plausible-sounding but nonsensical guidance.^57^ When five AI chatbots provided references that could answer medical questions, the rate of hallucination for relevance to the prompt keywords was 62%, and the proportion of wrong answers ranged from 10% to 85%.^58^ When 227 radiologists assessed 324 chest X-rays, their diagnostic accuracy improved when supported by an AI assistant if it appeared highly confident, but worsened when it was uncertain.^59^

Taken together, these studies show that LLMs and generative AI tools are not yet reliable enough to be integrated into clinical decision-making. Even when they are used to support searches for information, scrutiny of their responses should be mandatory. All LLMs are capable of some errors, which would not be a major concern if their errors were obvious, but LLMs can produce plausible answers that may be hard to detect. When challenged, AI assistants frequently admitted mistakes even if they had not made any.^29^ New AI tools are more likely to be implemented if they are trusted, so questions for further research should include how their linguistic styles may influence medical thinking.

## How should the medical community respond?

AI tools—and especially LLMs and general-purpose AI—are promoted heavily by companies whose primary motivation has become the maximization of returns for shareholders, not better public health. Some politicians embrace ‘AI’ enthusiastically and uncritically as a panacea that will boost economic growth. Software tools may be developed by engineers with little first-hand experience of the principles of scientific evidence and no experience of clinical practice or medical ethics. For medical applications, doctors and patients need to work with regulators to define best practice.

Medical advances and specialization have led to factual overload, so the focus in many universities has shifted to problem-based learning, educating students how to retrieve and evaluate information and undertake evidence-based practice. Increasingly, those tasks will involve the use of AI tools, so we will need to adopt strategies that can compensate for our innate anthropomorphic tendencies. We need to understand what we can do, and should do, that machines cannot. Metacognition will help us to design and interact with AI systems so that they enhance but do not replace clinical and experiential learning.^60^

We can employ language cautiously, guarding against the use of terms that are vague or that may have a negative influence on how we think. Different tools will all have some uncertainty or error, which should always be disclosed. Bayesian approaches have been recommended when reporting results of ML models.^61^ Predictive models that estimate the probability of an outcome should be reported with confidence intervals. Journal editors should insist that reports of studies using any type of AI include full disclosure of their imprecision and limitations.^62^

When language models are retrained on larger datasets, their performance can deteriorate.^63^ If hallucinations are not removed at source, there may be a risk that they will contaminate and corrupt future iterations when a model is retrained and upgraded.^27^ Gradual corruption or epistemic decay of the content of the web would lead to progressive erosion of trust and perhaps eventual ‘epistemic collapse’ when we can no longer tell what is true or judge what to believe. Scientific knowledge evolves, so it is essential that sources for any medical LLMs are carefully revised and updated. A recent comparison showed that smaller fine-tuned biomedical LLMs underperformed on medical tasks compared with general models^64^ so medical databases will need to be curated on a large cooperative scale.

### Regulation

Diagnostic and clinical-decision support systems that incorporate AI or ML are defined as medical devices, so they need regulatory approval. The evidence required for certification of any ‘software as a medical device’ (SaMD) should be proportionate to the risks related to its function and context of use,^25^ but guidance from regulators on appropriate methods for clinical evaluation is lacking. A review of 903 AI-enabled medical devices approved by the US Food and Drug Administration up to August 2024 found that 24% had not been evaluated by clinical performance studies, 44% had studies that were not reported publicly, only 8% of investigations had been prospective, and only 2% were randomized trials.^65^ Devices approved without clinical validation are 2.8 times more likely to be recalled.^66^

Clinical researchers should contribute to drafting regulatory guidance and common technical specifications.^67–69^ Claims for new AI- and ML-enabled medical devices must not be exaggerated. Methods are available that can improve the transparency of sources for LLMs (*Table 3*).^70–77^ The first LLM to receive formal approval and CE-marking as a Class IIb (moderate risk) medical device (in 2025) was ‘Prof Valmed’, which is a clinical decision-support tool that uses retrieval-augmented generation (RAG) and ChatGPT4.0 to search for answers within a curated database of guidelines and published literature. In a recent study, however, it did not perform better than a general LLM for suggesting rare rheumatological diagnoses.^78^

**Table 3:** Some methods that could increase the transparency and accuracy of LLMs

Solution	Reference	Definition
Model cards	Margaret Mitchell *et al.*, 2019^70^	Recommended framework for transparent reporting of the performance characteristics of any machine learning model, especially computer vision or natural language processing, aiming to minimize bias and standardize ethical practice.
Electronic watermarking	John Kirchenbauer *et al.*, 2023^71^	Embedding signals into generated text that are invisible to humans but algorithmically detectable from a short span of tokens. Serves as a means for predicting if text has been generated by a machine, by giving a statistical probability that it is synthetic.
Transclusion	Ted Nelson, quoted by Tim Berners-Lee, 2025^72^	Every time a document is quoted in another document, there should be a bidirectional link between the original and the quotation, allowing switching between them.
Chain of verification	Shehzaad Dhuliawala *et al.*, 2023^73^	This method was developed so that when an LLM drafts its initial response, it also creates questions to verify the answers, which are then compared before generating a final verified response.
Retrieval-augmented generation	Siru Liu *et al.*, 2025^74^ Can Wang *et al.*, 2026^75^	The accuracy of an LLM and the relevance of its response for a specific question can be increased by integrating additional external sources of data provided by the user.
Guardrails	Urs Gasser & Viktor Mayer-Schönberger, 2024^76^ Joe Hakim *et al.*, 2025^77^	Decisional guardrails refer to a general concept of setting acceptable boundaries at the interface between a person’s choice and the input of society. Guardrails mitigated certain types of hallucinations and errors from LLMs, for drug safety queries, with potential applicability to other medical safety-critical contexts.

## Anthropomorphism

The Greek word for man is anthropos (άνθρωπος), while morfi (μορφή) means form, and morfismos (μορφισμός) is a mathematical formulation that links or equates two terms. The etymology of anthropomorphism—as applied to animals—implies that they are considered to have properties that are like human ones. The noun has come to refer also to the misattribution of human characteristics to objects, but perhaps now it would be useful for emphasis to have a separate word that relates specifically to software.

One option might be to incorporate logos (λόγος) since its Greek meanings include word and reason, but logomorphism was used by Owen Barfield in 1928 to describe the action of philosophers who misinterpret past statements by ‘the fallacy of projecting their own consciousness onto others, usually older states of consciousness’.^79^ Mechanomorphism, technomorphism, and computationalism have all been used to denote the reverse (and similarly misguided) phenomenon of attributing machine characteristics to humans.

The Greek word for software is λογισμικό (logismikó). Assigning human characteristics to software could, in English, become ‘logismiko-anthropomorphism’, but that would be too long. With apologies to native speakers and scholars of Greek, the compound noun could be shortened to ‘logimorphism’ and defined as endowing software with human qualities.

## Discussion

Logimorphism, as a special case of anthropomorphism, may be dangerous. Many authors have emphasized risks that stem from an uncritical approach to the implementation of AI tools, and the misleading language that is now in common parlance when referring to software does not help. As summarized in this essay, AI is not intelligent, neural networks do not replicate connections in the human brain, and LLMs process numbers rather than words. In my opinion, LLMs and chatbots should not reply in the first person. Sycophancy should be outlawed.

As a general term, ‘AI’ is too broad. Software tools that use statistical methods for analyzing data or making probabilistic inferences should respond to instructions with factual statements that emphasize how their outputs have been determined. In science and medicine, we should distinguish clearly between well-validated ML algorithms that allow human oversight—which we can use safely and effectively—and generative AI or LLMs, where the balance is less certain and caution will always be indicated.^46^ Their allure is that they offer “the seductive promise of expertise without experience”^80^ but they remain mindless machines.

When Peirce recognized in 1887 that “the capacity of a machine has absolute limitations; it has been contrived to do a certain thing, and it can do nothing else”,^3^ he could have been predicting what is now called narrow AI. Fine-tuned models work well for highly defined tasks, but LLMs and generative AI have deficiencies that will persist; errors and hallucinations are inherent in their methodologies and therefore unavoidable.^24^ In some respects, LLMs plagiarize the data on which they were trained, yet they have been developed without a requirement to cite primary sources as would be mandatory for scientific reports.

Most LLMs are being updated and improved continuously, and new generative AI models are being developed for specific tasks, so critics may claim that the views in this commentary will rapidly become obsolete. Nonetheless, it will remain crucial to perform clinical studies for each new AI tool intended for a clinical application, to demonstrate if it makes fewer mistakes and reduces risks, since it is likely that those will never be eliminated.

Overconfidence in the veracity of outputs from AI tools may be similar to applying heuristics: reaching a premature conclusion without sufficient thought increases the chances of making a wrong decision. Very few studies, if any, have demonstrated reduced morbidity or mortality from using an AI tool in a prospective trial; surrogate metrics have been preferred.^81^ There is a risk that adoption of AI tools may lead to deskilling; for example, the rate of detecting colonic adenomas fell by 6% when physicians were assisted by AI.^82^ It is concerning that 45% of the responses from ChatGPT in one study were considered to be empathetic, compared to <5% of those from physicians.^83^ Sympathetic human contact and communication must remain at the heart of clinical practice.

Turing concluded his 1950 paper by stating that ‘We may hope that machines will eventually compete with men in all purely intellectual fields’.^5^ Even if that was attainable—and my understanding is that research in cognitive neuroscience implies that it is not—in my opinion it would be undesirable, especially from a medical perspective. Computationalism reduces human consciousness, thinking and intelligence to the level of computer operations, which grossly underestimates the capabilities of human beings. And if we could avoid logimorphism, by no longer talking about artificial ‘intelligence’ in medicine, it might help us to use new computing tools more wisely.^84^

## Data Availability

No new data were generated or analysed in support of this manuscript.

## References

[1] Lovelace A . Notes by the translator. [on Sketch of the Analytical Engine Invented by Charles Babbage Esq. by L.F. Menabrea] Note G. Scientific Memoirs Selected from the Transactions of Foreign Academies of Science and Learned Societies. London: Richard and John E. Taylor; 1843. p691.

[2] Babbage C . Chapter VIII, on the analytical engine. In: Passages from the Life of a Philosopher. London: Longman, Green, Longman, Roberts, & Green; 1864. p136.

[3] Peirce CS . Logical machines. Am J Psychology 1887;1:165–170.

[4] Turing AM . Computing machinery and intelligence. Mind 1950;59:433–460.

[5] McCarthy J, Minsky ML, Rochester N, Shannon CE. A proposal for the Dartmouth Summer Research Project on Artificial Intelligence. 1955. https://raysolomonoff.com/dartmouth/boxa/dart564props.pdf.

[6] Hayes P, Ford K. Turing test considered harmful. In: *Proceedings of the 14th International Joint Conference on Artificial Intelligence*. IJCAI 1995, pp. 972–977. https://www.ijcai.org/Proceedings/95-1/Papers/125.pdf.

[7] High-Level Expert Group on Artificial Intelligence . A definition of AI: main capabilities and scientific disciplines. European Commission. 2018. https://ec.europa.eu/futurium/en/system/files/ged/ai_hleg_definition_of_ai_18_december_1.pdf.

[8] Lekadir K, Quaglio G, Tselioudis Garmendia A, Gallin C. Artificial intelligence in healthcare. Applications, risks, and ethical and societal impacts. European Parliamentary Research Service - Scientific Foresight Unit. PE 729.512, 2022.

[9] Organisation for Economic Co-operation and Development . OECD Science, Technology and Industry Scoreboard 2017: the Digital Transformation. Paris: OECD Publishing; 2017.

[10] Russell S . Human Compatible. London: Allen Lane; 2019. P. 41.

[11] Glasser MF, Coalson TS, Robinson EC, Hacker CD, Harwell J, Yacoub E, et al A multi-modal parcellation of human cerebral cortex. Nature 2016;536:171–178. 27437579 10.1038/nature18933 PMC4990127

[12] Jung RE, Haier RJ. The parieto-frontal integration theory (P-FIT) of intelligence: converging neuroimaging evidence. Behav Brain Sci 2007;30:135–154. 17655784 10.1017/S0140525X07001185

[13] Fedorenko E, Ivanova AA, Regev TI. The language network as a natural kind within the broader landscape of the human brain. Nat Rev Neurosci 2024;25:289–312. 38609551 10.1038/s41583-024-00802-4 PMC13222024

[14] Tuckute G, Kanwisher N, Fedorenko E. Language in brains, minds, and machines. Annu Rev Neurosci 2024;47:277–301. 38669478 10.1146/annurev-neuro-120623-101142

[15] McCulloch WS, Pitts W. A logical calculus of the ideas immanent in nervous activity. 1943. Bull Math Biol 1990;52:99–115. [Reprinted from the Bulletin of Mathematical Biophysics. 1943;5:115–133]. 2185863

[16] Herculano-Houzel S . The human brain in numbers: a linearly scaled-up primate brain. Front Hum Neurosci 2009;3:31. 19915731 10.3389/neuro.09.031.2009 PMC2776484

[17] Zhang J . Basic neural units of the brain: neurons, synapses and action potential. 2019. arXiv:1906.01703.

[18] Pakkenberg B, Pelvig D, Marner L, Bundgaard MJ, Gundersen HJ, Nyengaard JR, et al Aging and the human neocortex. Exp Gerontol 2003;38:95–99. 12543266 10.1016/s0531-5565(02)00151-1

[19] Aru J, Larkum ME, Shine JM. The feasibility of artificial consciousness through the lens of neuroscience. Trends Neurosci 2023;46:1008–1017. 37863713 10.1016/j.tins.2023.09.009

[20] Penrose R . The Emperor’s New Mind: Concerning Computers, Minds, and the Laws of Physics. Oxford: Oxford University Press; 1989.

[21] Seth AK, Bayne T. Theories of consciousness. Nat Rev Neurosci 2022;23:439–452. 35505255 10.1038/s41583-022-00587-4

[22] Cogitate Consortium; Ferrante O, Gorska-Klimowska U, Henin S, Hirschhorn R, Khalaf A, et al Adversarial testing of global neuronal workspace and integrated information theories of consciousness. Nature 2025;642:133–142. 40307561 10.1038/s41586-025-08888-1 PMC12137136

[23] Bishop JM . Artificial intelligence is stupid and causal reasoning will not fix it. Front Psychol 2021;11:513474. 33584394 10.3389/fpsyg.2020.513474 PMC7874145

[24] Larson EJ . The Myth of Artificial Intelligence: why Computers Can’t Think the way we do. Cambridge, Massachusetts: The Belknap Press of Harvard University Press; 2021.

[25] Rademakers FE, Biasin E, Bruining N, Caiani EG, Davies RH, Gilbert SH, et al CORE-MD clinical risk score for regulatory evaluation of artificial intelligence-based medical device software. NPJ Digit Med 2025;8:90. 39915308 10.1038/s41746-025-01459-8 PMC11802784

[26] Boroditsky L . How language shapes thought. Sci Am 2011;304:62–65. 21319543 10.1038/scientificamerican0211-62

[27] Asadi M, O'Sullivan JW, Cao F, Nedaee T, Rajabalifardi K, Li FF, et al MIRAGE: the illusion of visual understanding. 2026. arXiv:2603.21687.

[28] Al-Dalakta A, Honnekeri B, Rodriguez F, Laffin L, Sunder V, Bruemmer D, et al Inaccurate information regarding cardiovascular disease prevention enabled by generative artificial intelligence. Am J Prev Cardiol 2026;25:101404. 41551752 10.1016/j.ajpc.2025.101404 PMC12811462

[29] Sharma M, Tong M, Korbak T, Duvenaud D, Askell A, Bowman SR, et al Towards understanding sycophancy in language models. 2025. arXiv:2310.13548.

[30] Cohen MK, Kolt N, Bengio Y, Hadfield GK, Russell S. Regulating advanced artificial agents. Science 2024;384:36–38. 38574134 10.1126/science.adl0625

[31] Askell A, Carlsmith J, Olah C, Kaplan J, Karnofsky H, several Claude models, and many other contributors. Claude’s Constitution. Anthropic. 2026. https://www-cdn.anthropic.com/d0636f72a9493d279ed36b33987da3430bcb5911/claudes-constitution_webPDF_26-02.02a.pdf (21 January 2026).

[32] Suzgun M, Gur T, Bianchi F, Ho DE, Icard T, Jurafsky D, et al Language models cannot reliably distinguish belief from knowledge and fact. Nat Mach Intell 2025;7:1780–1790.

[33] Ibrahim L, Hafner FS, Rocher L. Training language models to be warm can reduce accuracy and increase sycophancy. Nature 2026;652:1159–1165. 42056545 10.1038/s41586-026-10410-0 PMC13128435

[34] Omar M, Sorin V, Wieler LH, Charney AW, Kovatch P, Horowitz CR, et al Mapping the susceptibility of large language models to medical misinformation across clinical notes and social media: a cross-sectional benchmarking analysis. Lancet Digit Health 2026;8:100949. 41672646 10.1016/j.landig.2025.100949

[35] Dentella V, Günther F, Leivada E. Systematic testing of three language models reveals low language accuracy, absence of response stability, and a yes-response bias. Proc Natl Acad Sci U S A 2023;120:e2309583120. 38091290 10.1073/pnas.2309583120 PMC10743380

[36] Guan B, Roosta T, Passban P, Rezagholizadeh M. The order effect: investigating prompt sensitivity to input order in LLMs. 2025. arXiv:2502.04134v2.

[37] Du Y, Tian M, Ronanki S, Rongali S, Bodapati, Galstyan A, et al Context length alone hurts LLM performance despite perfect retrieval. 2025. arXiv:2510.05381.

[38] Steyvers M, Tejeda H, Kumar A, Belem AC, Karny S, Hu X, et al What large language models know and what people think they know. Nat Mach Intell 2025;7:221–231.

[39] Zhao JX, Liu JZJ, Hooi B, Ng SK. How does response length affect long-form factuality. 2025. arXiv:2505.23295.

[40] Wang W, Min J, Zou W. Intelligence degradation in long-context LLMs: critical threshold determination via natural length distribution analysis. 2026. arXiv:2601.15300.

[41] Weizenbaum J . Computer Power and Human Reason: From Judgment to Calculation. San Francisco: W. H. Freeman and Company; 1976.

[42] Stevens LM, Mortazavi BJ, Deo RC, Curtis L, Kao DP. Recommendations for reporting machine learning analyses in clinical research. Circ Cardiovasc Qual Outcomes 2020;13:e006556. 33079589 10.1161/CIRCOUTCOMES.120.006556 PMC8320533

[43] Faes L, Sim DA, van Smeden M, Held U, Bossuyt PM, Bachmann LM. Artificial intelligence and statistics: just the old wine in new wineskins? Front Digit Health 2022;4:833912. 35156082 10.3389/fdgth.2022.833912 PMC8825497

[44] Plana D, Shung DL, Grimshaw AA, Saraf A, Sung JJY, Kann BH. Randomized clinical trials of machine learning interventions in health care: a systematic review. JAMA Netw Open 2022;5:e2233946. 36173632 10.1001/jamanetworkopen.2022.33946 PMC9523495

[45] Szegedy C, Zaremba W, Sutskever I, Bruna J, Erhan D, Goodfellow I, et al Intriguing properties of neural networks. 2013. arXiv:1312.6199.

[46] Obuchowicz R, Piórkowski A, Nurzyńska K, Obuchowicz B, Strzelecki M, Bielecka M. Will AI replace physicians in the near future? AI adoption barriers in medicine. Diagnostics (Basel) 2026;16:396. 41681714 10.3390/diagnostics16030396 PMC12897247

[47] Tsai MJ, Lin PY, Lee ME. Adversarial attacks on medical image classification. Cancers (Basel) 2023;15:4228. 37686504 10.3390/cancers15174228 PMC10487122

[48] Smith G . The AI Delusion. Oxford: Oxford University Press; 2018.

[49] Hill BG, Koback FL, Schilling PL. The risk of shortcutting in deep learning algorithms for medical imaging research. Sci Rep 2024;14:29224. 39587148 10.1038/s41598-024-79838-6 PMC11589829

[50] Ben-Michael E, Greiner DJ, Huang M, Imai K, Jiang Z, Shin S. Does AI help humans make better decisions? A statistical evaluation framework for experimental and observational studies. Proc Natl Acad Sci U S A 2025;122:e2505106122. 40961147 10.1073/pnas.2505106122 PMC12478102

[51] Vaccaro M, Almaatouq A, Malone T. When combinations of humans and AI are useful: a systematic review and meta-analysis. Nat Hum Behav 2024;8:2293–2303. 39468277 10.1038/s41562-024-02024-1 PMC11659167

[52] O'Sullivan JW, Palepu A, Saab K, Weng WH, Amponsah DK, Cheng E, et al A large language model for complex cardiology care. Nat Med 2026;32:616–623. 41652123 10.1038/s41591-025-04190-9 PMC12920087

[53] Goh E, Gallo RJ, Strong E, Weng Y, Kerman H, Freed JA, et al GPT-4 assistance for improvement of physician performance on patient care tasks: a randomized controlled trial. Nat Med 2025;31:1233–1238. 39910272 10.1038/s41591-024-03456-y PMC12380382

[54] Omar M, Agbareia R, McGreevy J, Gorenshtein A, Charney AW, Sakhuja A, et al LLMs can do medical harm: stress-testing clinical decisions under social pressure. 2025. medRxiv 2025.11.25.25340972.

[55] Wu D, Haredasht FN, Maharaj SK, Jain P, Tran J, Gwiazdon M, et al First, do NOHARM: towards clinically safe large language models. 2025. arXiv:2512.01241v2.

[56] Wang L, Li J, Zhuang B, Huang S, Fang M, Wang C, et al Accuracy of large language models when answering clinical research questions: systematic review and network meta-analysis. J Med Internet Res 2025;27:e64486. 40305085 10.2196/64486 PMC12079073

[57] Birkun AA, Gautam A. Large language model (LLM)-powered chatbots fail to generate guideline-consistent content on resuscitation and may provide potentially harmful advice. Prehosp Disaster Med 2023;38:757–763. 37927093 10.1017/S1049023X23006568

[58] Aljamaan F, Temsah MH, Altamimi I, Al-Eyadhy A, Jamal A, Alhasan K, et al Reference hallucination score for medical artificial intelligence chatbots: development and usability study. JMIR Med Inform 2024;12:e54345. 39083799 10.2196/54345 PMC11325115

[59] Agarwal N, Moehring A, Rajpurkar P, Salz T. Combining human expertise with artificial intelligence: experimental evidence from radiology. National Bureau of Economic Research, Working Paper 31422. 2023, revised November 2025. http://www.nber.org/papers/w31422.

[60] Howley LD, Whelan AJ. From the World Wide Web to AI: why we must learn from our past to transform the future of medical education. Acad Med 2025;100:S30–S33. 40456173 10.1097/ACM.0000000000006103

[61] Seoni S, Jahmunah V, Salvi M, Barua PD, Molinari F, Acharya UR. Application of uncertainty quantification to artificial intelligence in healthcare: a review of last decade (2013-2023). Comput Biol Med 2023;165:107441. 37683529 10.1016/j.compbiomed.2023.107441

[62] Burnell R, Schellaert W, Burden J, Ullman TD, Martinez-Plumed F, Tenenbaum JB, et al Rethink reporting of evaluation results in AI. Science 2023;380:136–138. 37053341 10.1126/science.adf6369

[63] Shumailov I, Shumaylov Z, Zhao Y, Papernot N, Anderson R, Gal Y. AI models collapse when trained on recursively generated data. Nature 2024;631:755–759. 39048682 10.1038/s41586-024-07566-y PMC11269175

[64] Dorfner FJ, Dada A, Busch F, Makowski MR, Han T, Truhn D, et al Evaluating the effectiveness of biomedical fine-tuning for large language models on clinical tasks. J Am Med Inform Assoc 2025;32:1015–1024. 40190132 10.1093/jamia/ocaf045 PMC12089759

[65] Windecker D, Baj G, Shiri I, Kazaj PM, Kaesmacher J, Gräni C, et al Generalizability of FDA-approved AI-enabled medical devices for clinical use. JAMA Netw Open 2025;8:e258052. 40305017 10.1001/jamanetworkopen.2025.8052 PMC12044510

[66] Lee B, Kramer P, Sandri S, Chanda R, Favorito C, Nasef O, et al Early recalls and clinical validation gaps in artificial intelligence-enabled medical devices. JAMA Health Forum 2025;6:e253172. 40844774 10.1001/jamahealthforum.2025.3172 PMC12374217

[67] Fraser AG, Biasin E, Bijnens B, Bruining N, Caiani EG, Cobbaert K, et al., for the CORE–MD consortium. Artificial intelligence in medical device software and high-risk medical devices – a review of definitions, expert recommendations and regulatory initiatives. Expert Rev Med Devices 2023;20:467–491. 37157833 10.1080/17434440.2023.2184685

[68] Armoundas AA, Loscalzo J. Do world-wide policy initiatives for regulating health care related artificial intelligence safeguard the declaration of Helsinki? EClinicalMedicine 2026;92:103784. 41717587 10.1016/j.eclinm.2026.103784 PMC12914512

[69] Ong JCL, Ning Y, Collins GS, Bitterman DS, Beecy AN, Chang RT, et al International partnership for governing generative artificial intelligence models in medicine. Nat Med 2025;31:2836–2839. 40588674 10.1038/s41591-025-03787-4

[70] Mitchell M, Wu S, Zaldivar A, Barnes P, Vasserman L, Hutchinson B, et al Model cards for model reporting. In: *Proceedings of the Conference on Fairness, Accountability, and Transparency*, Atlanta, GA, USA; 29 January 2019. Association for Computing Machinery, New York. Pp. 220–229.

[71] Kirchenbauer J, Geiping J, Wen Y, Katz J, Miers I, Goldstein T. A watermark for large language models. 2023. arxiv.org/abs/2301.10226.

[72] Berners-Lee T . This is for Everyone. London: Macmillan; 2025. p98.

[73] Dhuliawala S, Komeili M, Xu J, Raileanu R, Li X, Celikyilmaz A, et al Chain-of-verification reduces hallucination in large language models. 2023. arXiv:2309.11495.

[74] Liu S, McCoy AB, Wright A. Improving large language model applications in biomedicine with retrieval-augmented generation: a systematic review, meta-analysis, and clinical development guidelines. J Am Med Inform Assoc 2025;32:605–615. 39812777 10.1093/jamia/ocaf008 PMC12005634

[75] Wang C, Chen Y. Evaluating large language models for evidence-based clinical question answering. Patterns (N Y) 2026;7:101519. 42130942 10.1016/j.patter.2026.101519 PMC13161685

[76] Gasser U, Mayer-Schönberger V. Guardrails Guiding Human Decisions in the age of AI. Princeton: Princeton University Press; 2024.

[77] Hakim JB, Painter JL, Ramcharran D, Kara V, Powell G, Sobczak P, et al The need for guardrails with large language models in pharmacovigilance and other medical safety critical settings. Sci Rep 2025;15:27886. 40738919 10.1038/s41598-025-09138-0 PMC12311179

[78] Kremer P, Langballe E, Haase I, Bamberger J, Kuhn S, Krusche M, et al Diagnostic performance of prof. Valmed, ChatGPT-5 thinking, and OpenEvidence in rheumatology: a comparative evaluation. Rheumatol Int 2026;46:31. 41518413 10.1007/s00296-025-06068-y PMC12790495

[79] Loftin L . Logomorphism: projecting modern consciousness onto ancient minds. Cosmos and History: The Journal of Natural and Social Philosophy 2024;20:320–341.

[80] Celi LA . Teaching machines to doubt. Nat Med 2025;31:3964. 41120765 10.1038/s41591-025-04013-x

[81] Muneer A, Zhang K, Hamdi I, Qureshi R, Waqas M, Fouad S, et al Foundation models in biomedical imaging: turning hype into reality. 2025. arXiv:2512.15808. 10.1038/s41551-026-01762-z 42595819

[82] Budzyń K, Romańczyk M, Kitala D, Kołodziej P, Bugajski M, Adami HO, et al Endoscopist deskilling risk after exposure to artificial intelligence in colonoscopy: a multicentre, observational study. Lancet Gastroenterol Hepatol 2025;10:896–903. 40816301 10.1016/S2468-1253(25)00133-5

[83] Ayers JW, Poliak A, Dredze M, Leas EC, Zhu Z, Kelley JB, et al Comparing physician and artificial intelligence chatbot responses to patient questions posted to a public social media forum. JAMA Intern Med 2023;183:589–596. 37115527 10.1001/jamainternmed.2023.1838 PMC10148230

[84] Cruz-Aguilar MA . The epistemic revolution of AI: reconfiguring the foundations of scientific knowledge. AI Soc 2026;41:2041–2057.

